# Study on sodium ion supplementation performance of CNT-coated sodium oxalate in sodium ion batteries

**DOI:** 10.1016/j.isci.2025.114581

**Published:** 2025-12-31

**Authors:** Shengdong Tao, Yanyan Xuan, Jian Li, Kewei Lei, Zulv Huang, Guowen He, Kun Shen, Zheng Liu, Zhifang Yin

**Affiliations:** 1 School of Materials and Chemical Engineering, Key Laboratory of Low Carbon and Environmental Functional Materials of College of Hunan province, Hunan City University, Yiyang 413000, China; 2 Hunan Zhengyuan Institute for Energy Storage Materials and Devices, Changsha 410083, Hunan, China; 3 School of Materials Science and Engineering, Central South University, Changsha 410083, Hunan, China

**Keywords:** electrochemistry, applied sciences, energy storage

## Abstract

Low initial coulombic efficiency of hard carbon anodes and irreversible sodium loss during cycling in sodium-ion batteries (SIBs) lead to reduced specific capacity and shortened cycle life. Introducing sodium supplementation can effectively enhance the specific capacity and cycle life of SIBs. Na_2_C_2_O_4_ was selected as the sodium compensation agent owing to its high theoretical capacity, excellent chemical stability, and environmental friendliness. To improve conductivity and reduce decomposition voltage of Na_2_C_2_O_4_, carbon nanotubes (CNTs) were employed for coating. The optimized 10% CNT-coated sodium oxalate (CNT-10@Na_2_C_2_O_4_) exhibited a reduced initial decomposition voltage of 3.82 V (vs. Na^+^/Na) and delivered an irreversible (sodium compensation) capacity of 392.65 mAh g^−1^. NNFMO-CN||HC full cells were assembled using NaNi_1/3_Fe_1/3_Mn_1/3_O_2_ cathode containing 10% CNT-10@Na_2_C_2_O_4_ and hard carbon anode, demonstrating a discharge retention capacity of 38.35 mAh g^−1^ after 100 cycles, higher than the retained capacity of only 15.16 mAh g^−1^ of the NNFMO||Na cell without CNT-10@Na_2_C_2_O_4_. These results indicate that CNT-10@Na_2_C_2_O_4_ significantly improves the specific capacity and cycling performance of SIBs.

## Highlights

• Efficiently synthesized CNT-10@Na_2_C_2_O_4_ with sodium compensation capacity of 392.11 mAh g^−1^
• CNT-10@Na_2_C_2_O_4_ improves cycle performance of battery (retains 38.35 mAh g^−1^ after 100 cycles)
• Provides a candidate for sodium compensation material of sodium-ion batteries

## Graphical abstract

**Figure:**
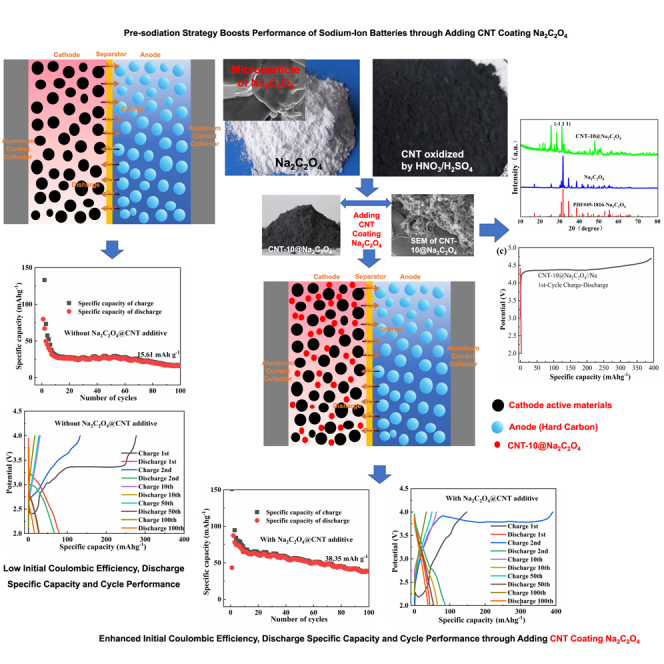

## Introduction

As a potential alternative to lithium-ion batteries, sodium-ion batteries (SIBs) have garnered significant attention due to the abundance of sodium resources, low cost, environmental friendliness, and high safety profiles.^1^^,^^2^^,^^3^^,^^4^ Hard carbon (HC), a promising anode material for SIBs, exhibits commercial viability owing to its wide availability of raw materials, high sodium storage capacity, and low production costs. However, structural defects on the surface and within HC materials severely degrade SIB performance. Surface defects induce continuous formation of the solid electrolyte interphase (SEI) layer on the surface of HC materials during cycling, leading to reduced initial coulombic efficiency (ICE) and specific capacity, which further affects (compromises) cycling stability and accelerates performance degradation over repeated charge-discharge cycles of SIBs.^4^^,^^5^^,^^6^^,^^7^^,^^8^ Repetitive cracking and restructuring of the SEI layer on HC surface continuously consumes limited sodium ions from cathode materials, further exacerbating capacity decay. Additionally, structural defects within HC materials can lead to irreversible sodium insertion, resulting in excessive sodium consumption, which significantly decreases the reversible capacity, energy density, and cycling performance of the battery, thereby affecting the commercial viability of SIBs.^9^^,^^10^^,^^11^^,^^12^^,^^13^

To address these issues, researchers have proposed various strategies, including interfacial regulation,^14^^,^^15^^,^^16^^,^^17^ electrolyte optimization, and sodium compensation techniques.^18^^,^^19^ While the first two approaches aim to mitigate sodium loss, the last focuses on compensating for sodium loss during the initial charging and subsequent cycle processes, emerging as a highly promising solution in recent years. Sodium compensation strategies can be categorized into physical methods, electrochemical methods, and additive-based pre-sodiation techniques for anodes or cathodes.^19^ Physical method typically employs metallic sodium, such as the sodium powder-based compensation technology developed by Tang,^20^ which improves the ICE and cycling performance of SIBs. However, the stringent moisture/oxygen-free operational requirements and uncontrollable sodium compensation processes render physical methods cost-prohibitive and technically challenging. Electrochemical method involves complexities related to battery disassembly and reassembly, limiting their scalability for commercial applications. In contrast, sodium supplementation additive-based approaches utilize sodium-rich compounds incorporated into electrodes or active materials, demonstrating superior practicality for industrial adoption.

Currently, numerous inorganic and organic sodium-containing compounds have been explored as cathode/anode additives for sodium compensation in SIBs. Anode sodium compensation additives typically consist of strongly reductive organic sodium salts, such as sodium naphthalene,^21^^,^^22^^,^^23^ sodium biphenyl,^24^^,^^25^ and sodium diphenyl ketone (benzophenone).^26^ These organic sodium salts, while capable of delivering sodium compensation at low potentials through oxidation processes, require inert atmosphere protection during synthesis due to their high reactivity with metallic sodium and organic compounds. Moreover, their complex pre-sodiation procedures exhibit limited compatibility with current battery manufacturing technologies. In contrast, cathode sodium compensation additives exhibit superior electrochemical stability with redox potentials intermediate between cathode and anode materials. Although primarily incorporated into cathodes, they have occasionally been utilized in specific anode systems. Currently investigated cathode sodium compensators include diethylenetriamine pentaacetate (DTPA-5Na),^27^ Na_2_S,^28^^,^^29^ Na_2_O_2_,^30^^,^^31^ Na_3_P,^32^^,^^33^ NaN_3_,^34^ trisodium citrate (C_6_H_5_O_7_Na_3_),^35^ oxygen-doped sodium sulfophosphate (thiophosphate) (Na_3_PS_3_O),^36^ sodium *p*-aminobenzoate (PABZ-Na),^37^ sodium alkoxide aluminum hydride (alkoxyaluminum hydride),^38^ sodium acetate,^39^^,^^40^ sodium oxalate,^41^ sodium carbonate,^42^^,^^43^ NaCrO_2_,^44^ and sodium oxalate.^45^ Compared with other sodium compensation additives, most cathode sodium compensation additives exhibit enhanced safety profiles, compatibility with electrode fabrication processes, controllable costs, and promising application prospects, thus attracting extensive research interest in recent years. However, the development of ideal sodium compensation additives simultaneously possessing high gravimetric/volumetric capacity, efficient capacity utilization, moderate decomposition voltage, non-toxicity, excellent thermal/chemical stability, facile processing compatibility, and low production costs remains challenging. Optimization and modification of existing additives to approach or achieve these characteristics represents a crucial pathway for developing high-performance sodium compensation additives.

From perspectives of capacity, cost, stability, and environmental friendliness, sodium oxalate (Na_2_C_2_O_4_) emerges as a promising sodium compensation additive with a theoretical capacity of 400 mAh g^−1^. However, its practical application is significantly hindered by inherent limitations, notably high decomposition voltage and material alkalinity-induced slurry gelation, which reduce compatibility with conventional cathode materials. To address these issues, strategies involving conductive agent blending and surface coating have been implemented. For instance, Sun^46^ developed a Na_2_C_2_O_4_/activated carbon (AC) composite electrode, where AC facilitated Na_2_C_2_O_4_ decomposition, achieving a specific capacity of 393.8 mAh g^−1^. Guo^45^ designed a composite of synthesized Na_2_C_2_O_4_ with conductive carbon (SP), significantly reduced its oxidation potential of Na_2_C_2_O_4_ from 4.41 V to 3.97 V, substantially enhancing its usability. Cao^19^ employed a low-temperature carbon coating-assisted ball-milling technique to markedly decrease the decomposition voltage of Na_2_C_2_O_4_ from 4.42 V to 3.91 V, simultaneously elevating the full-cell energy density from 135.0 Wh kg^−1^ to 236.4 Wh kg^−1^.

While these studies have provided valuable insights for commercializing Na_2_C_2_O_4_ as a sodium compensating agent, its decomposition voltage remains relatively high, and achieving complete decomposition of Na_2_C_2_O_4_ to release all Na^+^ still requires a higher operational voltage, posing challenges for practical implementation. Therefore, developing advanced strategies to effectively reduce the decomposition voltage of Na_2_C_2_O_4_ while alleviating its slurry gelation issues remains imperative. Carbon nanotubes (CNTs), recognized for their superior conductivity and large specific surface area, have been widely employed as conductive additives in battery field to enhance electronic conduction within electrodes, optimize conductive pathways, and improve overall electrical conductivity performance. Building on this foundation, this study proposes a strategy involving CNT coating of Na_2_C_2_O_4_ to enhance its conductivity. Through systematic investigation of the impact of CNT coating content on the sodium compensation performance of Na_2_C_2_O_4_ and detailed characterization of structural/morphological evolution, this work aims to elucidate the sodium compensation evolution mechanisms and performance variations and provide reference data during the sodium compensation process. It is anticipated that the conductivity of sodium oxalate will improve after coating, reducing the exposure of its surface and alleviating the challenges faced by Na_2_C_2_O_4_ as a sodium compensation additive. The CNT@Na_2_C_2_O_4_ composite material optimized and prepared based on this design is expected to significantly enhance the electrochemical performance of SIBs, contributing to the industrialization of SIBs.

## Results and discussion

### Morphological and structural analysis

**Figure 1:**
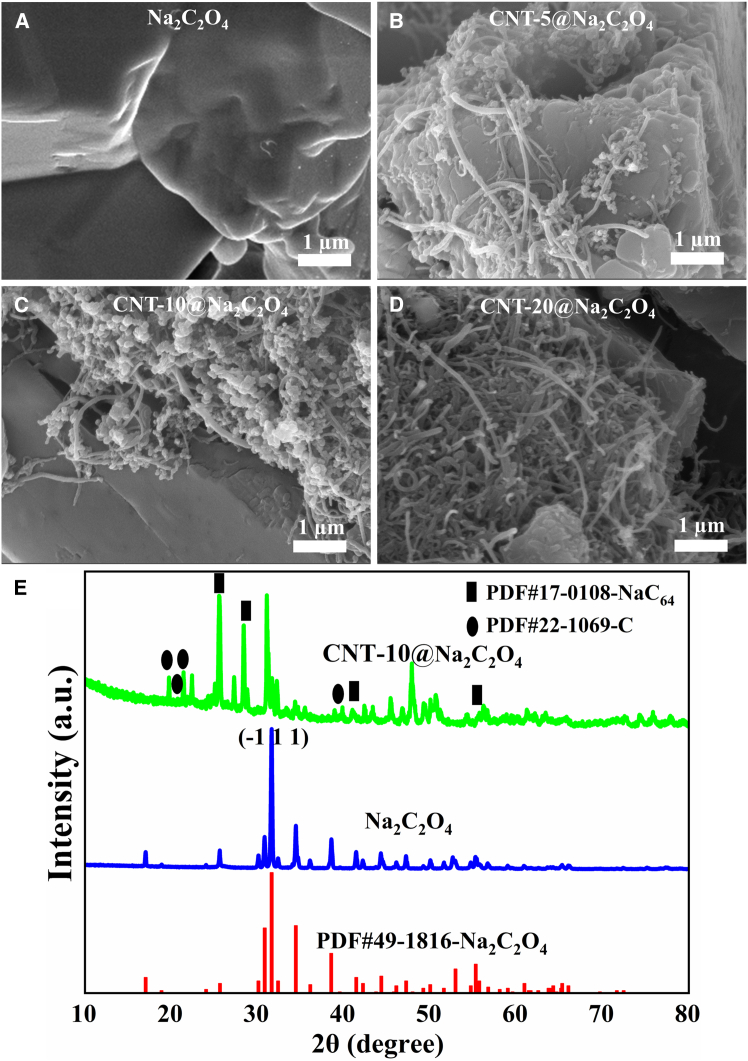
SEM images and XRD patterns (A) Na_2_C_2_O_4_ SEM image, (B), (C), (D) SEM images of CNT-5, 10, 20@ Na_2_C_2_O_4_, (E) XRD spectrum of Na_2_C_2_O_4_ and CNT-10@ Na_2_C_2_O_4_.

To investigate the morphological and structural evolution of sodium oxalate before and after CNT coating, scanning electron microscopy (SEM) and X-ray diffraction (XRD) characterizations were performed on sodium oxalate and sodium oxalate coated with 10% CNT (CNT-10@Na_2_C_2_O_4_), as shown in Figure 1. Figures 1A–1D present the SEM images of Na_2_C_2_O_4_, CNT-5@Na_2_C_2_O_4_, CNT-10@Na_2_C_2_O_4_, and CNT-20@Na_2_C_2_O_4_. Na_2_C_2_O_4_ (Figure 1A) exhibits irregular micro-sized particles with smooth surfaces and no coating layer. As shown in Figure 1B, most Na_2_C_2_O_4_ particles surface is mostly exposed, with only a small amount of linear CNT coating in CNT-5@Na_2_C_2_O_4_, indicating a poor coating effect (Figure S1B). At 10% CNT content, the surface of the sodium oxalate particles is mostly coated with CNT, forming a porous coating layer (Figure 1C). In Figure 1D, the coating layer on sodium oxalate is thicker, and the surface of Na_2_C_2_O_4_ is difficult to observe due to excessive CNT coating layer on Na_2_C_2_O_4_ particles (Figure S1D). The CNT conductive network (Figures S1B–S1D) on the surface of CNT@Na_2_C_2_O_4_ is expected to enhance electrical conductivity of Na_2_C_2_O_4_ through effective electron transport pathways. Upon the decomposition of Na_2_C_2_O_4_ within CNT@Na_2_C_2_O_4_ during cycling, the residual CNT framework (Figures S4B–S4D) maintains structural integrity at the composite sites, thereby preventing electrode collapse and ensuring mechanical stability. Notably, excessive CNT coating (20%, Figure S1D) induces a dense coating layer that potentially obstructs Na^+^ diffusion channels in CNT-20@ Na_2_C_2_O_4_, thereby compromising the sodium compensation performance of CNT-20@Na_2_C_2_O_4_.

XRD patterns in Figure 1E demonstrate that sodium oxalate matches well with the standard monoclinic phase (PDF#49-1816), exhibiting its strongest diffraction peak at 31.6° corresponding to the (−1 1 1) crystallographic plane. CNT-10@Na_2_C_2_O_4_ retains most characteristic peaks of Na_2_C_2_O_4_ with attenuated intensity, accompanied by emerging peaks from the CNT coating. The diffraction peaks at 25.43° and 28.59° are assigned to the NaC_64_ phase (PDF#17-0108). The diffraction peaks at 19.85°, 20.84°, and 21.55° are assigned to the carbon phase (PDF#22-1069) in the CNTs. During the oxidative treatment with concentrated sulfuric and nitric acids, oxygen-containing functional groups were introduced onto the CNT surfaces. When these functionalized CNTs were blended with the sodium oxalate solution, partial interaction between sodium ions and the oxygenated sites led to the formation of a NaC_64_ phase. As a result, the CNT-10@Na_2_C_2_O_4_ composite comprises not only crystalline Na_2_C_2_O_4_ (PDF#49-1816) and carbon from CNTs (PDF#22-1069), but also the newly formed NaC_64_ phase (PDF#17-0108). The emergence of this NaC_64_ phase is anticipated to strengthen the interfacial adhesion between sodium oxalate and the CNTs, thereby enhancing the structural integrity of the composite coating. Although there is preservation of the crystal structure of sodium oxalate after CNT coating, the appearance of diffraction peaks of the NaC_64_ and CNT coating (Figure 1C) causes some of the sodium oxalate diffraction peaks to be diminished. The coating of NaC_64_ and CNT is expected to facilitate electron and sodium ion transport during desodiation, which would contribute to improved electrical conductivity and electrochemical properties of sodium oxalate matrix, ultimately boosting its sodium compensation capability in energy storage applications.

### Electrochemical performance analysis of CNT@Na_2_C_2_O_4_

**Figure 2:**
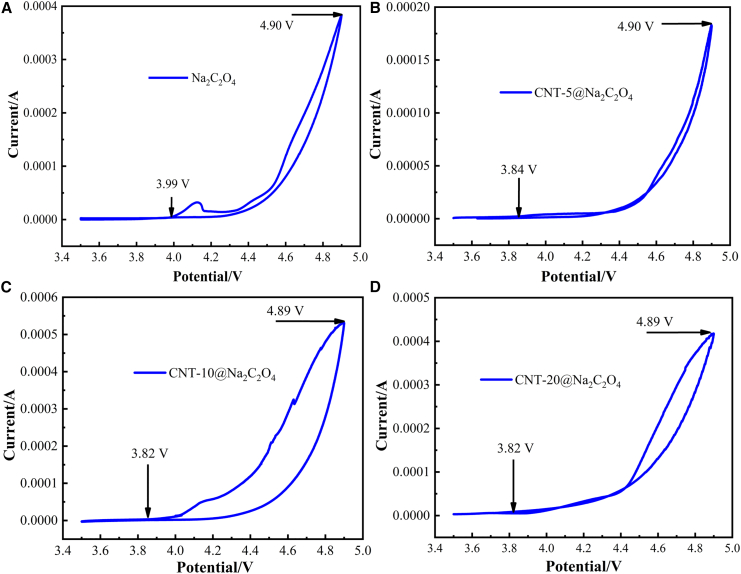
CV curves of CNT@Na_2_C_2_O_4_ with different CNT contents (A) Na_2_C_2_O_4_, (B) CNT-5@Na_2_C_2_O_4_, (C) CNT-10@Na_2_C_2_O_4_, (D) CNT-20@Na_2_C_2_O_4_ at 0.2 mV s^−1^.

The cathodes fabricated with Na_2_C_2_O_4_ and different CNT@Na_2_C_2_O_4_ composites were assembled into Na_2_C_2_O_4_||Na and CNT@Na_2_C_2_O_4_||Na cells. Figure 2 presents the cyclic voltammetry (CV) curves of Na_2_C_2_O_4_ and different CNT@Na_2_C_2_O_4_ composites at the scan rate of 0.2 mV s^−1^; the initial and maximum decomposition voltages were derived from these CV profiles. As shown in Figures 2A–2D, all CV curves exhibited a closed loop with voltage scanning from 3.5 V to ∼4.9 V (current peak) before returning to 3.5 V. Notably, only oxidation peaks without corresponding reduction peaks were observed in Figure 2, indicating the irreversible electrochemical oxidation decomposition of sodium oxalate. This irreversibility implies that the deintercalated Na^+^ ions cannot be reincorporated into the host structure during reduction, leading to irreversible structural degradation.

In Figure 2A, sodium oxalate begins to decompose at 3.99 V and the decomposition voltage reaches a peak current at 4.90 V (Figure S2A). In contrast, CNT-5@Na_2_C_2_O_4_ (Figures 2B and S2B) exhibited a lower-onset decomposition voltage of 3.84 V with a peak at 4.90 V, CNT-10@Na_2_C_2_O_4_ (Figures 2C and S2C) and CNT-20@Na_2_C_2_O_4_ (Figures 2D and S2D) demonstrated initial decomposition voltages of 3.82 V, both achieving peak currents at ∼4.89 V. These results reveal that the initial decomposition voltage of CNT@Na_2_C_2_O_4_ gradually decreased with increasing CNT content and stabilized when the CNT content exceeded 10%, with the CNT-10@Na_2_C_2_O_4_ having the lowest initial decomposition voltage of 3.82 V. This phenomenon can be attributed to the enhanced electrical conductivity achieved through the integration of highly conductive and linear CNTs. The pristine sodium oxalate consists of large, irregular particles with poor conductivity, whereas the CNT network facilitates charge transfer, thereby promoting material decomposition and Na^+^ extraction at lower potentials.

### Sodium compensation capacity analysis

**Figure 3:**
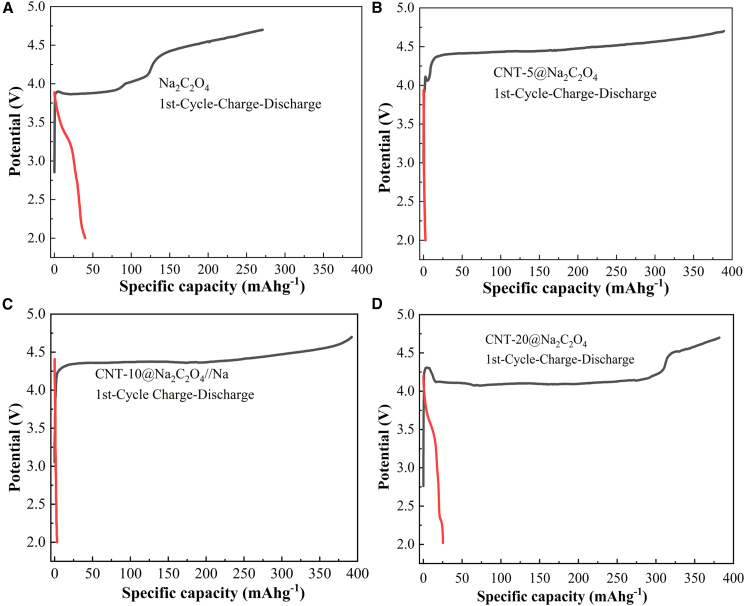
First charge-discharge curves of CNT@Na_2_C_2_O_4_ with different CNT contents (A) Na_2_C_2_O_4_, (B) CNT-5@Na_2_C_2_O_4_, (C) CNT-10@Na_2_C_2_O_4_, (D) CNT-20@Na_2_C_2_O_4_.

**Figure 4:**
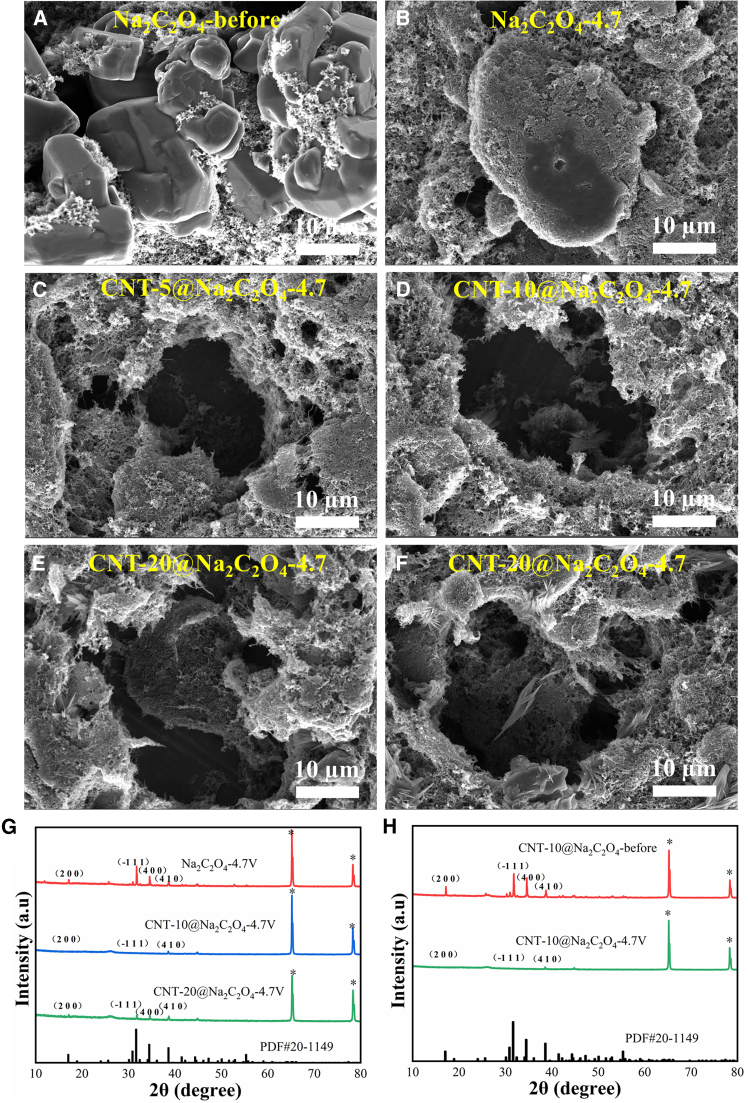
SEM images and XRD patterns (A) Na_2_C_2_O_4_ cathode before cycle; (B–F) Na_2_C_2_O_4_, CNT-5@Na_2_C_2_O_4_, CNT-10@Na_2_C_2_O_4_, and CNT-20@Na_2_C_2_O_4_ cathode electrodes after cycling at 4.7 V; (G) XRD patterns of Na_2_C_2_O_4_, CNT-10@Na_2_C_2_O_4_, and CNT-20@Na_2_C_2_O_4_ cathode electrodes after cycling at 4.7 V; (H) XRD patterns of CNT-10@Na_2_C_2_O_4_ cathode electrodes before and after cycling at 4.7 V.

Galvanostatic charge-discharge tests of Na_2_C_2_O_4_||Na and CNT@Na_2_C_2_O_4_||Na cells were performed within a voltage window of 2.0–4.7 V at 0.1 C. Figure 3 and Table 1 exhibit the initial charge-discharge profiles, specific capacities, and irreversible capacity losses of these cells. Figure 4 presents the SEM images and XRD patterns of pristine Na_2_C_2_O_4_ electrodes before and after cycling (2.0–4.7 V), as well as CNT-coated sodium oxalate electrodes post cycling.

**Table 1:** First charge-discharge capacities of pristine Na_2_C_2_O_4_ and CNT@Na_2_C_2_O_4_

Different Sodium Supplement	First Charge specific capacity/mA h g^−1^	First Discharge specific capacity/mA h g^−1^	Irreversible Capacity/mA h g^−1^
Na_2_C_2_O_4_	270.77	39.77	231.00
CNT-5@Na_2_C_2_O_4_	389.23	2.38	386.85
CNT-10@Na_2_C_2_O_4_	392.11	3.18	388.93
CNT-20@Na_2_C_2_O_4_	381.78	25.19	356.59

As illustrated in Figure 3 and Table 1, the pristine Na_2_C_2_O_4_ electrodes demonstrate irregular charge profiles lacking defined plateaus, accompanied by parasitic reactions below 4.0 V, yielding the lowest initial charge specific capacity (270.77 mAh g^−1^). CNT-coating modifications markedly improve electrochemical behavior, with charge capacities increasing alongside smoother voltage profiles and distinct charge plateaus. Notably, the CNT-10@Na_2_C_2_O_4_ composite demonstrates optimal performance, delivering a maximum charge specific capacity of 392.11 mAh g^−1^ with plateau-dominated charge curves and suppressed side reactions. In contrast, excessive CNT loading (20 wt %) induces capacity reduction (356.59 mAh g^−1^) and reappearance of non-plateau features in the charge curve, indicative of kinetic limitations (increasing semicircle radius in Figure S5D). The 2^nd^ and 3^rd^ charge-discharge capacities of Na_2_C_2_O_4_ and CNT@Na_2_C_2_O_4_ are extremely low (Figure S3). Corresponding irreversible capacities are quantified as 231.00, 386.85, 388.93, and 356.59 mAh g^−1^ for pristine Na_2_C_2_O_4_, CNT-5@ Na_2_C_2_O_4_, CNT-10@ Na_2_C_2_O_4_, and CNT-20@ Na_2_C_2_O_4_, respectively.

**Figure 5:**
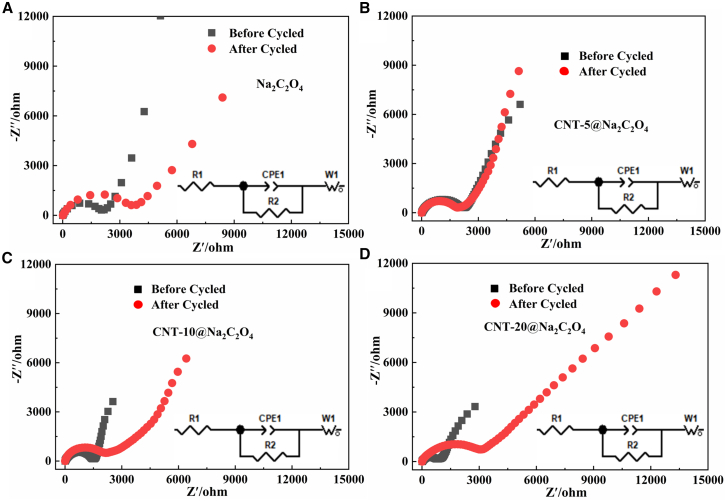
Nyquist curves (A) Na_2_C_2_O_4_||Na, (B) CNT-5@Na_2_C_2_O_4_||Na, (C) CNT-10@Na_2_C_2_O_4_||Na, and (D) CNT-20@Na_2_C_2_O_4_||Na cells.

The inferior charge specific capacity of pristine Na_2_C_2_O_4_ primarily results from the limitations of uncoated Na₂C₂O₄ particles, which lack a conductive CNT network. As shown in Figure 1A, such particles exhibit several critical drawbacks: inherently poor electrical conductivity, a high decomposition voltage (>4.7 V), and partial dissolution due to the absence of CNT encapsulation. Direct exposure to the electrolyte further induces surface side reactions, leading to poorly defined charge plateaus and low charge/reversible capacity. In contrast, CNT coating effectively reduces particle size and establishes conductive pathways (Figures 1B–1D). The enhanced electronic conductivity lowers the effective decomposition voltage, facilitating more complete Na^+^ extraction at 4.7 V. Progressive improvement in CNT coating quality (from 5% to 10%) optimizes charge-transfer kinetics as indicated by the smaller semicircle radii in EIS spectra (Figures S5B and S5C). Consequently, CNT-10@Na₂C₂O₄ delivers optimal performance, with near-theoretical capacity utilization and stable charge plateaus. However, excessive CNT coating (20 wt %) introduces thick conductive layers that impede Na⁺ diffusion kinetics, evidenced by the decreased slope in Figure 5D. This leads to incomplete decomposition and partial side reactions, thereby reducing charge specific capacity and distorting the charge profile with non-plateau features. These results confirm that CNT-10@Na_2_C_2_O_4_ enables the most complete Na^+^ extraction during cycling, simultaneously maximizing both charge specific capacity and irreversible capacity.

Figures 4A–4F, 4G, and 4H present the SEM images and XRD patterns of CNT-coated Na_2_C_2_O_4_ electrodes with varying CNT loadings before and after cycling at 4.7 V, respectively. As shown in Figures 4A–4F, the CNT-10@ Na_2_C_2_O_4_ electrode exhibits significant void formation and no discernible Na_2_C_2_O_4_ particles after cycling. Correspondingly, the XRD pattern (Figure 4H) displays near-complete disappearance of Na_2_C_2_O_4_ characteristic diffraction peaks compared with the pre-cycled state, confirming minimal residual Na_2_C_2_O_4_ and near-total sodium ion extraction. Conversely, electrodes with 0% and 20% CNT coatings retain partial Na_2_C_2_O_4_ particles (Figure S4) post cycling, as substantiated by residual (−1 1 1) diffraction peaks at 31.6° (∗ denotes Al current collector artifacts) in their XRD patterns.

These observations further validate that optimal CNT coating (10 wt %) establishes a continuous conductive network on Na_2_C_2_O_4_ surfaces while reducing particle size (Figures 1B–1D), synergistically enhancing sodium ion extraction kinetics and accelerating Na_2_C_2_O_4_ decomposition. Conversely, excessive CNT loading (20 wt %) induces overlapping conductive layers that impede sodium ion diffusion, resulting in kinetic limitations, partial decomposition, and reduced capacities. CNT-10@ Na_2_C_2_O_4_ achieves maximum charge specific capacity (392.11 mAh g^−1^) and irreversible capacity (388.93 mAh g^−1^). Therefore, 10% CNT coating is identified as the optimal CNT coating content, balancing enhanced conductivity with favorable ion transport kinetics to maximize electrochemical performance.

### Electrochemical impedance analysis after cycling

Figure 5 systematically compares the electrochemical impedance spectra (EIS) of pristine Na_2_C_2_O_4_ and CNT-coated Na_2_C_2_O_4_ composites with varying CNT loadings before and after charge-discharge cycling. Nyquist plots (Figure 5) exhibit a high-frequency semicircle corresponding to interfacial resistance and a low-frequency Warburg tail reflecting charge transfer resistance. The corresponding equivalent circuits and extracted impedance parameters are provided in Table 2.

**Table 2:** Fitted impedance values of batteries fabricated with different CNT@Na_2_C_2_O_4_, before and after cycling

Impedance Name	Na_2_C_2_O_4_		CNT-5@Na_2_C_2_O_4_		CNT-10@Na_2_C_2_O_4_		CNT-20@Na_2_C_2_O_4_	
	Before Cycle	After Cycle	Before Cycle	After Cycle	Before Cycle	After Cycle	Before Cycle	After Cycle
R_1_	5.0	11.7	6.3	4.9	6.8	5.6	6.8	6.3
R_2_	1910	3808	2244	1689	1478	1981	794	2776
Wo-R	3111	8097	2760	3290	1094	6142	1469	6944

From Figure 5, before cycling, it can be observed that, with the increase of CNT coating, the radius of the semicircle gradually decreases (Figure S5), while the slope of the line exhibits irregular fluctuations. After cycling, the radius of the semicircle gradually decreases first and then increases (Figure S5), while the slope of the line increases first and then decreases. The shrinking semicircle radius indicates reduced charge transfer resistance, whereas the fluctuating slope suggests the variation of ion diffusion kinetics.

According to Table 2, the R_1_ values before cycling fall within a narrow range of 5–7 Ω, indicating consistency and minimal variation across samples. After cycling, the R_1_ value of the uncoated Na_2_C_2_O_4_ increased markedly to 11.7 Ω, whereas the CNT-coated samples exhibited negligible change, indicating superior cycled stability of CNT-coated samples. In terms of charge transfer resistance (R_2_), CNT-10@Na_2_C_2_O_4_ and CNT-20@Na_2_C_2_O_4_ exhibited a lower initial value before cycling. Post cycling, R_2_ increased for uncoated Na_2_C_2_O_4_, CNT-10@Na_2_C_2_O_4_ and CNT-20@Na_2_C_2_O_4_. In contrast, R_2_ decreased for CNT-5@Na_2_C_2_O_4_ after cycling. These results suggest that an optimal CNT coating effectively reduces the charge transfer resistance during and after cycling, thereby facilitating electron transfer and sodium ion extraction.

With the coating of sodium oxalate by CNT, the ion diffusion resistance (Wo-R) prior to cycling initially decreases and then increases. When the CNT coating amount is 10%, CNT-10%@Na_2_C_2_O_4_ exhibits the lowest ion diffusion resistance. After cycling, the Warburg diffusion resistance increases for all materials. This indicates that an appropriate CNT coating can effectively enhance the ion diffusion kinetics of sodium oxalate, promoting sodium ion migration and diffusion. However, an excessively thick coating layer (e.g., CNT-20%@Na_2_C_2_O_4_) increases ion diffusion resistance, thereby limiting the efficient transport of sodium ions.

In summary, an optimal CNT coating (e.g., in CNT-10@Na_2_C_2_O_4_) effectively reduces the charge transfer resistance during and after cycling and enhances the initial ion diffusion kinetics of sodium oxalate before cycling, thereby facilitating electron transfer and sodium ion extraction and sodium ion migration and diffusion. Although CNT coating reduces the internal charge transfer resistance of sodium oxalate, excessive CNT coating decreases ion diffusion kinetics (CNT-20@Na_2_C_2_O_4_) and hinders the release of sodium ions, causing ion diffusion impedance of the coated material to show a downward trend. Among the measured materials, CNT-10@Na_2_C_2_O_4_ exhibits the most favorable combination of low and stable charge transfer resistance before and after cycling, along with the lowest diffusion resistance after cycling. This material is therefore expected to deliver superior charge transport performance and enhanced sodium ion extraction capability.

### Performance of CNT-10@Sodium oxalate in full cell

**Figure 6:**
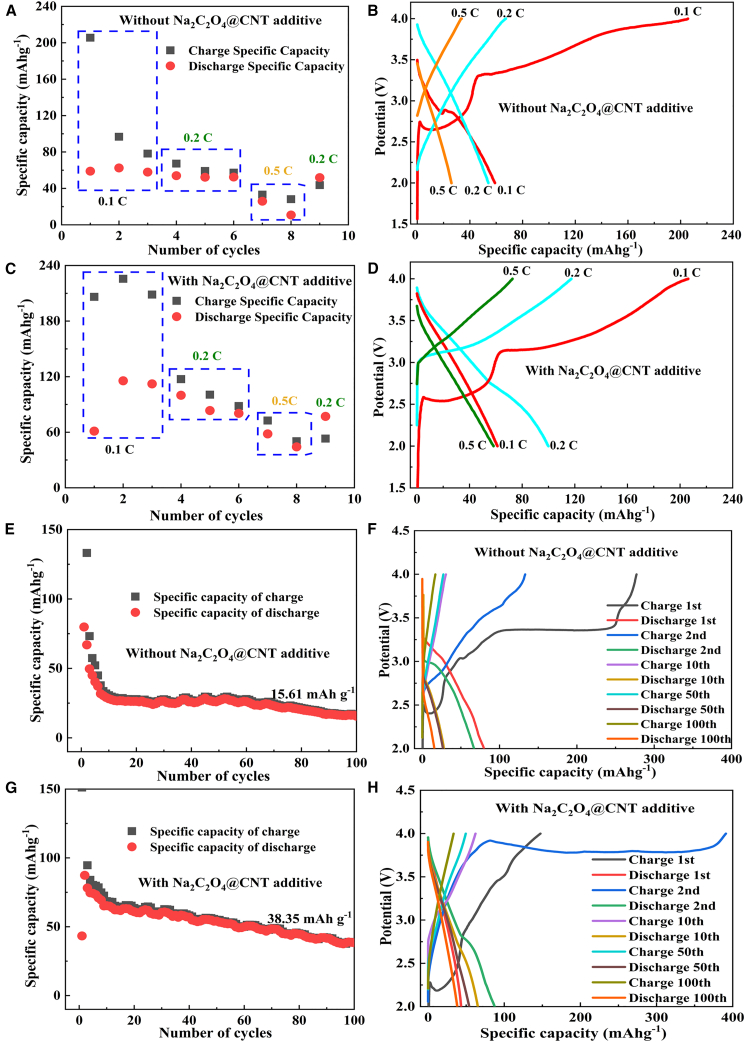
Cycling performance at different rates (A, B) Specific capacity and charge-discharge curves of the NNFMO||HC cell at different rates; (C, D) specific capacity and charge-discharge curves of the NNFMO-CN||HC cell at different rates; (E, F) specific capacity and charge-discharge curves of the NNFMO||HC cell at different cycle numbers; (G, H) specific capacity and charge-discharge curves of the NNFMO-CN||HC cell at different cycle numbers.

**Figure 7:**
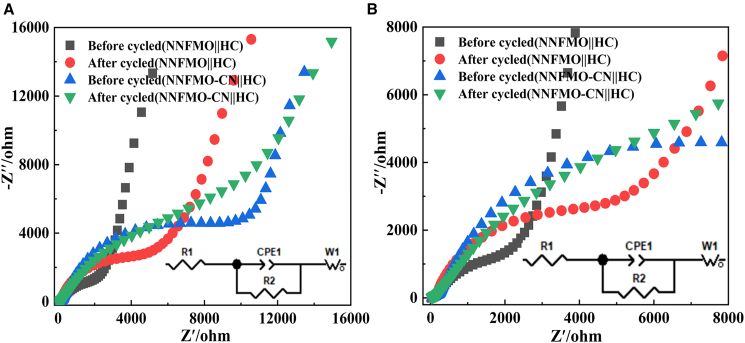
Nyquist curves (A) Before and after of NNFMO||HC and NNFMO-CN||HC cells; (B) partial enlarged view of (A).

Figure 6 illustrates the galvanostatic specific capacity and charge-discharge profiles of NNFMO||HC and NNFMO-CN (with 10% CNT-10%@sodium oxalate)||HC coin cells at different current rates, with the detailed charge-discharge specific capacity data presented in Tables 3, 4, and 5. Figure 7 presents the EIS of NNFMO||HC and NNFMO-CN||HC cells before and after cycling. As shown in Figures 6A–6D, the NNFMO-CN||HC cell demonstrates charge capacities of 206.18 mAh g^−1^, 117.35 mAh g^−1^, and 72.63 mAh g^−1^ at current densities of 0.1 C (1^st^ cycle), 0.2 C (4^th^ cycle), and 0.5 C (7^th^ cycle), respectively, with corresponding discharge capacities of 61.22 mAh g^−1^, 99.94 mAh g^−1^, and 58.22 mAh g^−1^ (Figure S6B; Table 3). In contrast, the NNFMO||HC cell shows charge capacities of 205.56 mAh g^−1^, 67.28 mAh g^−1^, and 33.22 mAh g^−1^ at the same current densities, with discharge capacities of 58.97 mAh g^−1^, 53.99 mAh g^−1^, and 25.96 mAh g^−1^ (Figures 6A, 6B, and S6A; Table 3). At a 0.1 C rate, both cells display low ICEs, with negligible capacity differentiation.

**Table 3:** Rate charging specific capacity of NNFMO||HC and NNFMO-CN|HC full cell

Specific Capacity at Different C-Rates	Charging Capacity at 0.1 C/mAh g^−1^	Charging Capacity at 0.2 C/mAh g^−1^	Charging Capacity at 0.5 C/mAh g^−1^	Discharging Capacity at 0.1 C/mAh g^−1^	Discharging Capacity at 0.2 C/mAh g^−1^	Discharging Capacity at 0.5 C/mAh g^−1^
NNFMO-CN||HC	206.18	117.35	72.63	61.22	99.94	58.22
NNFMO||HC	205.56	67.28	33.22	58.97	53.99	25.96

**Table 4:** Charge-discharge specific capacity of NNFMO||HC full cell

Cycle number	1	2	10	50	100
Specific Capacity of Charge/mAh g^−1^	277.14	133.16	30.65	27.28	17.01
Specific Capacity of Discharge/mAh g^−1^	79.87	67.00	28.19	26.72	15.61

**Table 5:** Charge-discharge specific capacity of NNFMO-CN||HC full Cell

Cycle number	1	2	10	50	100
Specific Capacity of Charge/mAh g^−1^	151.11	394.05	68.63	55.13	38.79
Specific Capacity of Discharge/mAh g^−1^	43.35	87.35	65.31	54.04	38.35

The low ICE is attributed to high-surface-area and over-capacitized HC anode, which promotes continuous parasitic reactions at the anode/electrolyte interface. This accelerates the formation of thick and inhomogeneous SEI, as reflected by the increased interface impedance after cycling, resulting in irreversible sodium ion loss during the initial cycle. This effect is further exacerbated in coin cells, which inherently possess a larger electrode-electrolyte interfacial volume compared with industrial 18650 batteries. The resulting weaker mechanical coupling and suboptimal interfacial contact between cathode and anode contribute to the large interfacial impedance shown in Figure 7. During the first cycle, irreversible Na^+^ loss from the NNFMO-based cathode occurs via two pathways: (1) electrochemical reduction of electrolyte components on the HC anode surface, which consumes active Na^+^ for SEI formation, and (2) Na⁺ intercalation into disordered graphitic domains of HC lattice, where steric hindrance and strong binding energies prevent their extraction during discharge. Notably, approximately 70% of cyclable Na^+^ is irreversibly consumed in SEI-related processes during the first cycle, as confirmed by the significant disparity between initial charge (205–207 mAh g^−1^) and discharge capacities (58–62 mAh g^−1^). Furthermore, the decreases in the slope of the corresponding line after cycling indicates slowed ion diffusion, consistent with irreversible Na⁺ loss after cycling (Figure 7).

At 0.1C, the low initial charge-discharge specific capacity of the NNFMO-CN||HC coin cell arises from the thick dual conductive architecture of Na_2_C_2_O_4_ additive coated by CNT and carbon black. This reduces the sodium ion migration rate in the supplement, causing ineffective extraction of sodium ions during the initial charge-discharge cycle at low current densities. As a result, the sodium supplementation performance of the NNFMO-CN||HC cell is not evident during the first cycle. However, the charge-discharge capacity of the cell increased significantly during the subsequent 2nd and 3rd cycles, higher than the NNFMO||HC cell (Figures 6A–6D and S6); the sodium supplementation performance was evident confirmed. At 0.2 C, the NNFMO-CN||HC cell demonstrates a substantial capacity enhancement charge and discharge specific capacity of 50.07 mAh g^−1^ and 45.95 mAh g^−1^ compared with the NNFMO||HC cell. With incremental gains of 39.41 mAh g^−1^ (charge) and 32.26 mAh g^−1^ (discharge) at 0.5 C. Critically, the NNFMO-CN||HC cell achieves superior rate discharge capability (99.94 mAh g^−1^ at 0.2 C, 58.22 mAh g^−1^ at 0.5 C) and enhanced coulombic efficiency (85.16% vs. 80.25% at 0.2 C, 80.16% vs. 78.15% at 0.5 C) after 0.1C cycles. Overall, the NNFMO-CN||HC cell exhibits significantly higher charge and discharge capacities and improved charge-discharge efficiency, validating the efficacy of sodium compensation strategies.

This enhancement is attributed to electrochemical activation during charge-discharge cycles at 0.1 C (1^st^ cycle) and enables progressive extraction of Na^+^ from the CNT-10@sodium oxalate reservoir at 0.1C (2^nd^ and 3^rd^ cycles) and 0.2 C (4^th^ cycle), replenishing anode deficiencies and elevating reversible capacity (Figures 6C, 6D, and S6B). Furthermore, the pre-formed SEI film of the 1^st^ cycle effectively passivates the anode/electrolyte interface, suppressing parasitic reactions, reducing irreversible Na^+^ consumption and improving charge-discharge efficiency.

Figures 6E–6H illustrate the specific capacity and charge-discharge curves of the NNFMO-CN||HC and NNFMO||HC coin cells at different cycle numbers, with detailed discharge capacities provided in Tables 4 and 5. As summarized in these data, NNFMO||HC exhibits rapid capacity degradation during cycling. The charge capacities progressively decline from 277.14 mAh g^−1^ (1^st^ cycle) to 17.01 mAh g^−1^ (100^th^ cycle), while the corresponding discharge capacities plummet from 79.87 mAh g^−1^ (1^st^ cycle) to 15.61 mAh g^−1^ (100^th^ cycle). In stark contrast, the NNFMO-CN||HC full cell demonstrates charge capacities of 151.11 mAh g^−1^, 394.05 mAh g^−1^, 68.63 mAh g^−1^, 55.13 mAh g^−1^, and 38.79 mAh g^−1^ at the 1^st^, 2^nd^, 5^th^, 10^th^, 50^th^, and 100^th^ cycles, respectively, with corresponding discharge capacities of 43.35 mAh g^−1^, 87.35 mAh g^−1^, 65.31 mAh g^−1^, 54.04 mAh g^−1^, and 38.35 mAh g^−1^ at the 1^st^, 2^nd^, 5^th^, 10^th^, 50^th^, and 100^th^ cycles.

Notably, after 100 cycles, discharge specific capacity of the NNFMO||HC coin cell retains merely 15.61 mAh g^−1^, whereas the NNFMO-CN||HC coin cell maintains 38.35 mAh g^−1^ capacity retention, highlighting the critical role of sodium supplementation in mitigating capacity fade and enhancing cyclability. This indicates that the addition of CNT@NaC_2_O_4_ supplement decouples SEI compensation from cathode degradation, suppressing the rapid consumption of Na^+^ in the cathode, slows down the rapid degradation of the capacity, thereby improving its cycling performance.

Intriguingly, the NNFMO-CN||HC cell exhibits anomalously low charge-discharge capacities during the initial cycle, followed by a sharp capacity surge in the 2^nd^ cycle (Figures 6C–6F). This phenomenon is mechanistically attributed to the enhanced thicker coating layer such as carbon black and CNT on the surface of NaC_2_O_4_, which kinetically impedes sodium-ion extraction (smaller line slope of the NNFMO-CN||HC cell before cycling, Figure 7; Table S1) during the first cycle and partially blocks electrolyte penetration, preventing full activation of sodium ions from both the cathode and NaC_2_O_4_. As cycling progresses, the electrolyte gradually infiltrates the CNT network and electrode pores, enabling progressive release of trapped sodium ions from the CNT-coated NaC_2_O_4_ and cathode. This delayed activation mechanism effectively replenishes sodium ions consumed by the HC anode, thereby triggering the abrupt capacity escalation in cycle 2 and sustaining enhanced cyclic stability.

Overall, parasitic side reactions between electrolyte components and electrode surfaces during charging elevate the practical charge specific capacity beyond the theoretical capacity (Figure 6). The cells exhibit transient capacity maxima during the initial cycles (Figures 6C–6F), mechanistically linked to the CR2032 coin-cell configuration incorporating dual separators (thick/thin layers), which establish multilayer separator-electrode interfaces influencing electrolyte wetting dynamics and SEI formation on the HC anode. Post 100 cycles, the discharge specific capacity of the NNFMO||HC cell remains 15.61mAh g^−1^(Figure 6C), whereas the discharge specific capacity of the NNFMO-CN||HC cell sustains 38.35 mAh g^−1^ (Figure 6D), significantly higher than that of the NNFMO||HC cell, indicating the efficacy of sodium supplementation in stabilizing long-term cyclability.

This performance enhancement arises from CNT@NaC_2_O_4_ as a controlled sodium-ion reservoir, progressively releasing Na^+^ to offset SEI-induced sodium loss at the HC anode during cycling, thus improving the cycling performance of the sodium-supplemented full battery. Concurrently, the CNT-reinforced coating on sodium oxalate provides good stability for the coating layer of sodium oxalate and offers supporting roles, improving the stability of the positive electrode material. On the other hand, CNT-reinforced coating enhances the material’s resistance to chemical stresses during charging and discharging and reduces degradation and structural damage. Electrochemical buffering through conductive CNT networks homogenize charge distribution and reduce localized overpotentials. Such structural-electrochemical synergy enhances interfacial contact integrity between active materials and NaC_2_O_4_, improving the cycle property of NNFMO-CN||HC.

In conclusion, the synergistic interplay between CNT-mediated mechanical stability and sodium-supplement-driven ion replenishment establishes a self-compensating electrochemical framework, achieving 38.35 mAh g^−1^ capacity retention in the NNFMO-CN||HC cell versus 15.61 mAh g^−1^ in the NNFMO||HC cell. This strategy pioneers a materials-design paradigm to address intrinsic sodium-loss challenges in post-lithium-ion battery systems.

To address the irreversible sodium loss originating from the low ICE of HC anodes in SIBs, this study proposes CNT-coated sodium oxalate as a novel self-sacrificial cathode additive for active sodium compensate. Electrochemical characterization reveals that the 10% CNT-coated sodium oxalate initiates oxidative decomposition at 3.82 V vs. Na^+^/Na during charging, delivering a charge specific capacity of 392.11 mAh g^−1^, with 388.93 mAh g^−1^ irreversible capacity (sodium compensation capacity). When implemented in full-cell configurations using NNFMO cathodes, HC anodes, and 10% CNT-10%@sodium oxalate (NNFMO-CN||HC), the cells exhibit significantly higher discharge specific capacity at 0.2 C and 0.5 C rates compared with the full-cell configurations using NNFMO cathodes and HC anodes (NNFMO||HC). Notably, the NNFMO-CN||HC cell maintains 38.35mAh g^−1^ capacity after 100 cycles at 0.2 C, representing a 2.5-fold enhancement compared with the NNFMO||HC cell (15.16 mAh g^−1^). This work establishes CNT-coated sodium oxalate as a cost-effective, eco-friendly, and safety-enhanced sodium compensation agent, providing new insights into cathode additive engineering for overcoming ICE limitations in SIBs. The demonstrated performance improvements highlight sodium oxalate’s potential as a viable sodium supplement for next-generation energy storage systems.

### Limitations of the study

While the study demonstrates the potential of CNT-10@Na_2_C_2_O_4_ in enhancing the performance of SIBs, it is important to acknowledge several limitations primarily arising from experimental constraints. First, the assessment of CNT-10@Na_2_C_2_O_4_ was conducted mainly in half-cell configurations. Full-cell performance, which is the ultimate benchmark for practical application, could not be comprehensively evaluated due to limitations in our commercial battery assembly and testing equipment. Consequently, key metrics for full cells, such as actual capacity retention under long-term cycling, rate capability under high current densities, and energy density, were not fully characterized. These partial data may affect the overall persuasiveness and thoroughness of the evidence presented regarding the impact of CNT-10@Na_2_C_2_O_4_ in realistic battery system. Furthermore, the scale of electrode preparation and cell assembly was limited to laboratory scale, which might introduce inconsistencies not present in industrial-scale manufacturing processes. We are going to focus on addressing these limitations by upgrading our device fabrication conditions to enable the assembly and testing of high-quality full cells in future work. More accurate and convincing evaluation of the long-term cycling stability, rate performance, and practical viability of our pre-sodiation strategy for SIB are to follow.

## Resource availability

### Lead contact

Requests for further information and resources should be directed to and will be fulfilled by the lead contact, Shengdong Tao (taoshendgong@hncu.edu.cn).

### Materials availability

All unique/stable reagents generated in this study are available from the lead contact with a completed materials transfer agreement.

### Data and code availability

• Data: All data reported in this paper are available from the lead contact upon reasonable request. Requests for further information and resources should be directed to and will be fulfilled by the lead contact, Shengdong Tao (taoshendgong@hncu.edu.cn).
• Code: No code was generated in the study.
• Other: Materials and protocols are available from the corresponding author upon request. Requests for further information and resources should be directed to and will be fulfilled by the lead contact, Shengdong Tao (taoshendgong@hncu.edu.cn).

## Acknowledgments

This study was financed (supported) by the 10.13039/501100001809National Natural Science Foundation of China (52102243), Youth 10.13039/501100004735Natural Science Foundation of Hunan Province of China (2023JJ40106), and Excellent Youth Foundation of Hunan Educational Committee (23B0735).

## Author contributions

S.T., as the first author and corresponding author, was responsible for experimental design, data collection, data analysis, drafting the initial manuscript, securing funding support, and corresponding author duties.

Y.X., as the second author, assisted with experiments, data collection, and preliminary data analysis and contributed to writing parts of the manuscript.

J.L. provided overall research supervision and guidance, offered methodological support, performed experimental operations, conducted data verification and quality control, and revised the technical content of the manuscript.

K.L. participated in investigations, assisted with experiments and data acquisition, and contributed to data verification or resource provision.

Z.H. was responsible for visualization, data management and archiving, technical support, literature review and background research, and manuscript proofreading.

G.H. provided overall research supervision and guidance, coordinated and allocated resources, set up the experimental environment, assisted with experiments or manuscript editing, and performed language polishing of the manuscript.

K.S. provided experimental resources and data analysis support, participated in formal analysis, and contributed to project management and funding acquisition.

Z.L., as a corresponding author, was responsible for supervision, manuscript review, structural design and revision, funding support, and overall coordination.

Z.Y. provided senior guidance, project supervision, and funding support and conducted the final review and approval of the manuscript.

## Declaration of interests

The authors declare that they have no known competing financial interests or personal relationships that could have appeared to influence the work reported in this paper.

## STAR★Methods

### Key resources table

**Table:** 

REAGENT or RESOURCE	SOURCE	IDENTIFIER
**Chemicals, peptides, and recombinant proteins**		

Chemicals,	Not applicable	Not applicable
Carbon nanotube (CNT)	Aladdin	C139883-50g, >90%, Inner Diameter: 5–10 nm, Outer Diameter: 10–30 nm, Length: 10–30 μm
nitric acid	China National Pharmaceutical Group Chemical Reagent Co., Ltd.	10014518 AR, 65.0–68.0%500 mL
sulfuric acid	China National Pharmaceutical Group Chemical Reagent Co., Ltd.	10021618 AR, 95.0–98.0%,500 mL
Sodium oxalate (Na_2_C_2_O_4_	Aladdin	S112348-500g, AR
ethanol	64-17-5 Innochem G00006	G00006, AR, 95%
NaNi_1/3_Fe_1/3_Mn_1/3_O_2_ cathode	Hunan Meite New Materials Technology Co., Ltd.	Battery Grade
Poly(vinylidene fluoride) (PVDF)	Innochem	A08946
acetylene black (super P Li)	Yantai Jiayineng New Materials Co., Ltd. (TIMCAL)	Battery Grade
1-Methyl-2-pyrrolidinone (NMP)	Aladdin	M100588-500 mL AR, ≥99%(GC)
Hard Carbin (HC)	BTR New Material Group Co., Ltd.	Battery Grade
peptides, and recombinant proteins(No)	–	–

### Experimental model and study participant details

Omitted as our study does not involve biological models.

### Method details

Detailed descriptions of all experimental procedures and analyses are provided as followed.

#### Prepared and characterization of CNT-Coated sodium oxalate

First, carbon nanotubes (CNTs) were oxidized by treatment with a concentrated nitric acid and sulfuric acid mixture (3:1 v/v), followed by centrifugation, washing, and drying to obtain oxidized CNTs. Subsequently, five portions of 2 g Na_2_C_2_O_4_ were each dissolved in 100 mL of deionized water under stirring for 1 h. Separately, 0.041 g, 0.105 g, 0.222 g, 0.353 g, and 0.500 g of oxidized CNTs were dispersed in 100 mL of ethanol under stirring for 1 h, followed by ultrasonication for 0.5 h to ensure homogeneous dispersion. The CNT dispersions with varying mass ratios were then uniformly mixed with the sodium oxalate solution under ultrasonication for 1 h, and stirred at 80 °C until complete evaporation.

The mixtures were stirred at 80 °C until complete solvent evaporation, followed by drying at 80°C for 12 h and grinding to obtain CNT-coated sodium oxalate composites with CNT mass ratios of 2%, 5%, 10%, 15%, and 20%, denoted as CNT-2@Na_2_C_2_O_4_, CNT-5@Na_2_C_2_O_4_, CNT-10@Na_2_C_2_O_4_, CNT-15@Na_2_C_2_O_4_, and CNT-20@Na_2_C_2_O_4_, respectively. The phase structure and morphological characteristics of the composites materials were analyzed by X-ray diffraction (XRD) and scanning electron microscopy (SEM).

#### Battery assembly and electrochemical testing

Cathode slurries were prepared by mixing CNT-@Na_2_C_2_O_4_ (with varying CNT contents CNT-coating Na_2_C_2_O_4_), or NaNi_1/3_Fe_1/3_Mn_1/3_O_2_ cathode (NNFMO) containing with 10% CNT-10@Na_2_C_2_O_4_, HC, polyvinylidene fluoride (PVDF), and acetylene black (conductive agent) in a mass ratio of 8:1:1 using N-methyl-2-pyrrolidone (NMP) as the solvent. The slurries were coated onto aluminum foils and dried at 70°C, followed by punching into 14 mm diameter electrodes. Sodium metal, HC, CNT-@Na_2_C_2_O_4_, NNFMO and NNFMO with 10% CNT-10@Na_2_C_2_O_4_ cathodes (NNFMO-CN) were assembled into assembled into CNT-@Na_2_C_2_O_4_||Na, NNFMO||HC anode, and NNFMO-CN||HC coin cells (CR2025-type) in an argon-filled glovebox (H_2_O< 0.01 ppm, O_2_<0.01 ppm). NNFMO-CN||HC cells were assembled using NaNi_1/3_Fe_1/3_Mn_1/3_O_2_ cathode containing with 10% CNT-10%@Na_2_C_2_O_4_ and HC anode. These coin cells were subjected to galvanostatic charge-discharge tests charge-discharge tests at 2.0∼4.7 V and 2.0∼4.0 V for cycles, cyclic voltammetry (CV) at 0.1 mV s^-1^ (3.5∼4.9 V), and electrochemical impedance spectroscopy (EIS, 0.01 Hz∼10^6^ Hz). Post-cycling analyses, structural and morphological changes of materials were characterized by XRD and SEM.

### Quantification and statistical analysis

No statistical methods, sample sizes, and software was used generated in the study.

### Additional resources

Not applicable as this is not a clinical trial.

There is no reference cited in the STAR Methods.

No figures or complex tables are embedded in the STAR Methods.
