# Supplementary material for: Study on sodium ion supplementation performance of CNT-coated sodium oxalate in sodium ion batteries
Source: iScience. 2025 Dec 31;29(2):114581. doi: 10.1016/j.isci.2025.114581 (PMC12855588; doi:10.1016/j.isci.2025.114581)
Supplement: Document S1. Figures S1–S6 and Table S1 [file mmc1.pdf]

iScience, Volume *29*

Supplemental information

Study on sodium ion supplementation performance of CNT-coated sodium oxalate in sodium ion batteries

Shengdong Tao, Yanyan Xuan, Jian Li, Kewei Lei, Zulv Huang, Guowen He, Kun Shen, Zheng Liu, and Zhifang Yin

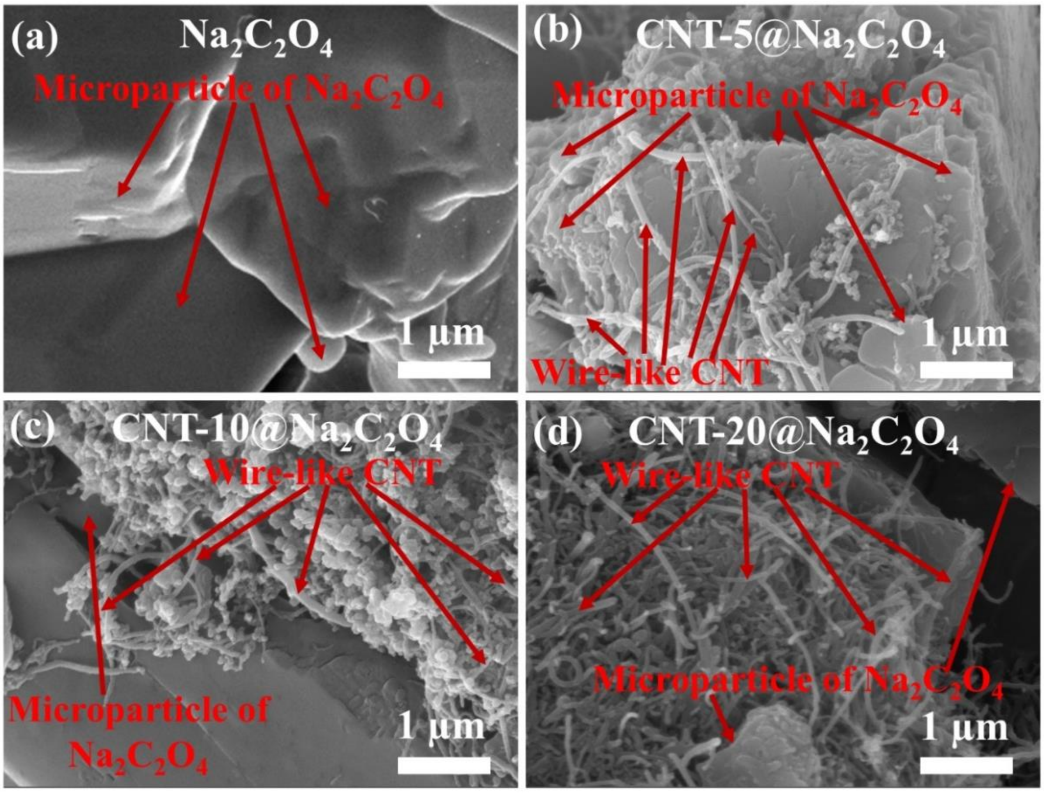

Figure S1 SEM images: (a) $Na_2C_2O_4$, (b) CNT-5@$Na_2C_2O_4$, (c) CNT-10@ $Na_2C_2O_4$, (d) CNT-20@$Na_2C_2O_4$

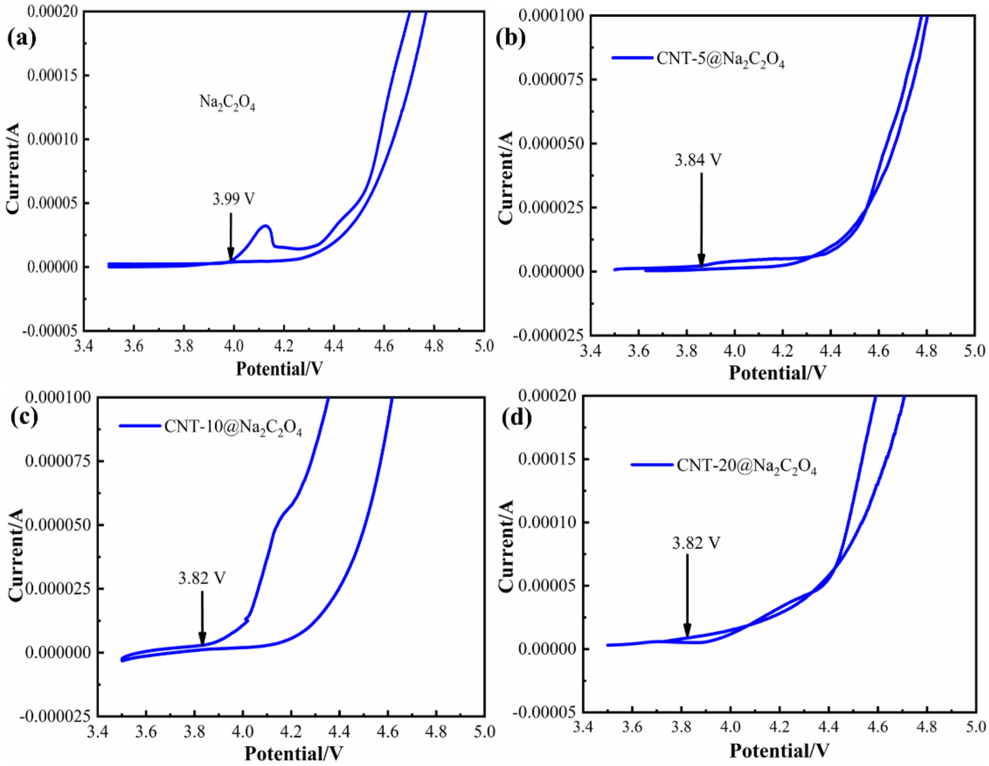

Figure S2 Partial enlarged view CV curves of CNT@$Na_2C_2O_4$ with different CNT contents: a $Na_2C_2O_4$, b, CNT-5@$Na_2C_2O_4$, c CNT-10@$Na_2C_2O_4$, d CNT-20@$Na_2C_2O_4$

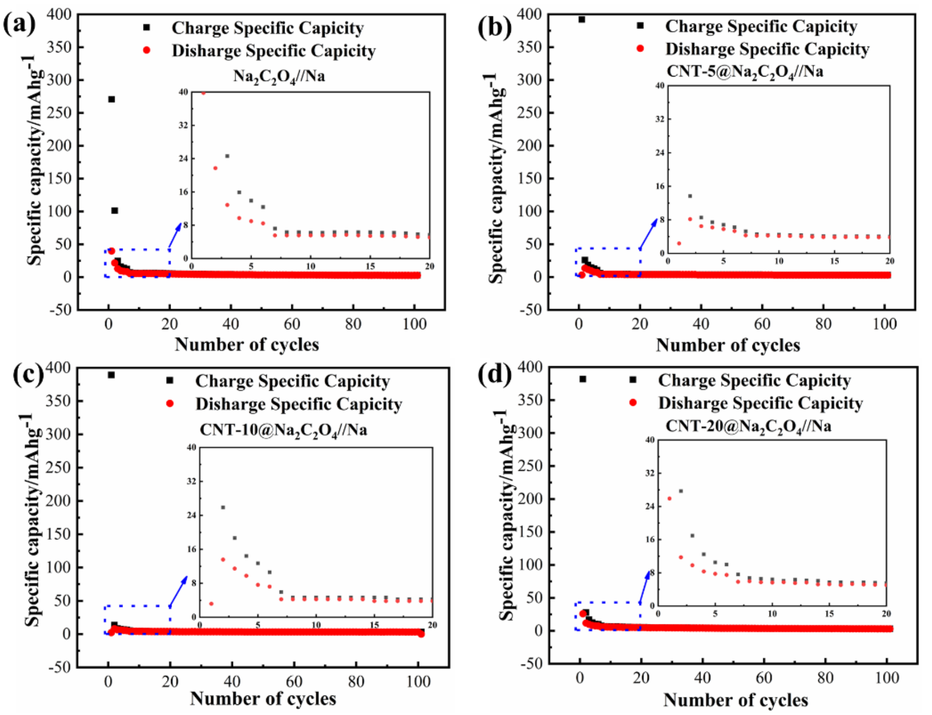

Figure S3 Cycling Specific capacity at different cycles numbers: (a)$Na_2C_2O_4$//Na, (b) CNT-5@$Na_2C_2O_4$//Na, (c) CNT-10@$Na_2C_2O_4$//Na, (d) CNT-20@$Na_2C_2O_4$//Na

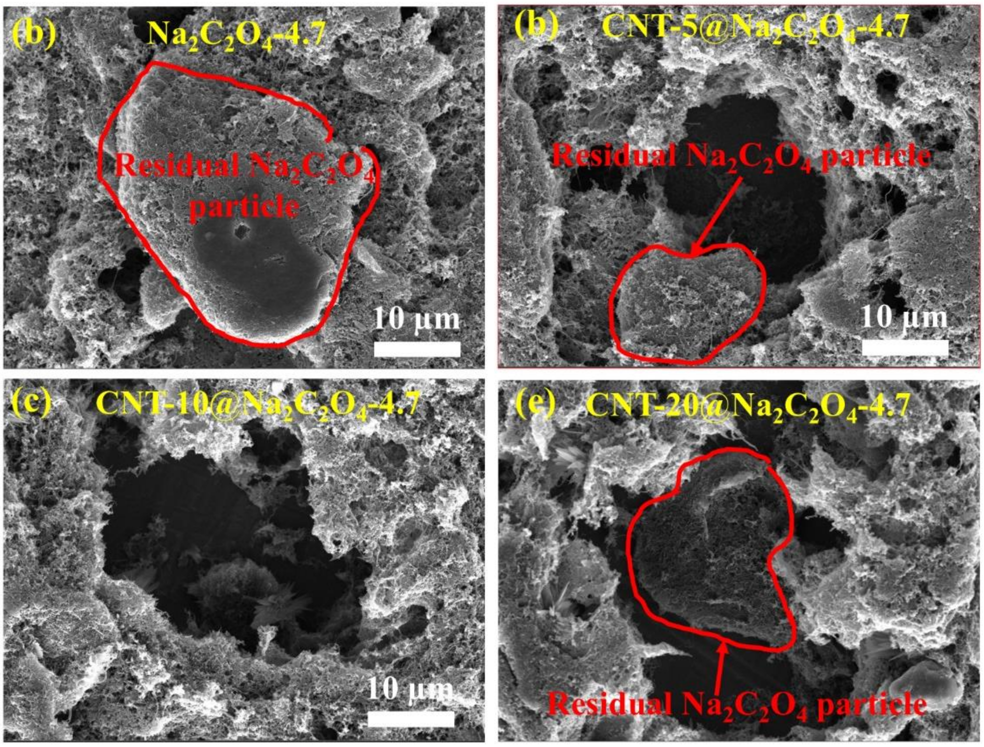

Figure S4 SEM images of after cycle: (a) $Na_2C_2O_4$, (b) CNT-5@$Na_2C_2O_4$, (c) CNT-10@$Na_2C_2O_4$, (d) CNT-20@$Na_2C_2O_4$

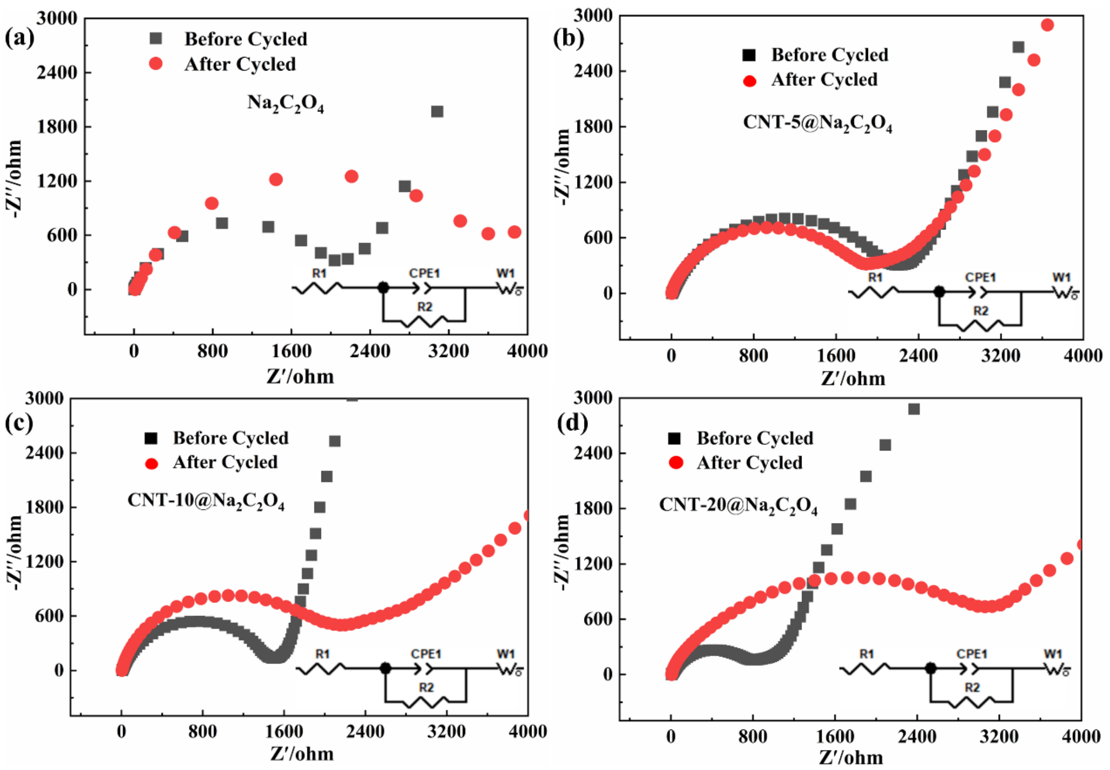

Figure S5 Partial enlarged view of Nyquist curves: (a) $Na_2C_2O_4$||Na, (b)CNT-5@$Na_2C_2O_4$||Na, CNT-10@$Na_2C_2O_4$||Na and CNT-20@$Na_2C_2O_4$||Na cells

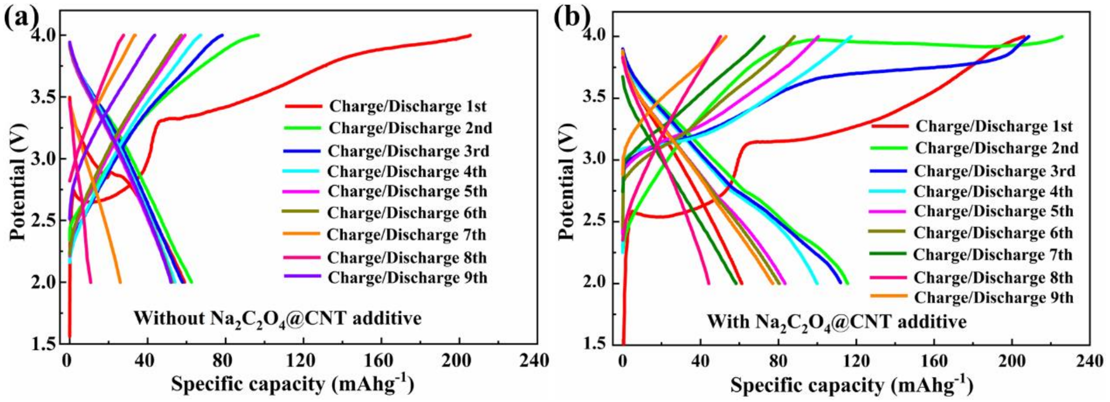

Figure S6 Charge-discharge curves of cells at different rates (a) NNFMO//HC cell, (b) NNFMO-CN//HC cell

Table S1 Fitted impedance values of batteries fabricated with different CNT@$Na_2C_2O_4$, before and after cycling

Impedance	NNFMO||HC		NNFMO-CN||HC	
Name	Before Cycle	After Cycle	Before Cycle	After Cycle
**R_1**	27.1	37.4	43.6	36.0
**R_p**	246.9	126.8	221.0	173.1
**Wo-R**	389.1	295.2	509.5	540.3
